# A Novel Level I Oncoplastic Surgery Technique for Tumors Located in UIQ of the Breast Far from the Nipple: The “Cross” Technique

**DOI:** 10.1097/GOX.0000000000002269

**Published:** 2019-07-26

**Authors:** Ahmad Kaviani, Sanaz Zand, Amir Ashraf-Ganjouei, Rami Younan, Remy Jacques Salmon

**Affiliations:** From the * Department of Surgery, Tehran University of Medical Sciences, Tehran, Iran; † Department of Surgery, University of Montreal, Montreal University Hospital Center (CHUM), Montreal, Montreal, Quebec, Canada; ‡ Department of Research, Kaviani Breast Disease Institute, Tehran, Iran; § Department of Breast Surgery, Les Peupliers Clinic, Paris, France; and (add new symbol) Breast Cancer Research Center, Tehran University of Medical Sciences, Tehran, Iran.

## Abstract

Breast surgery was revolutionized with the use of oncoplastic reshaping techniques minimizing breast deformities and esthetic complications. However, the application of the current oncoplastic techniques becomes challenging in some situations such as small-size breasts and when the tumors are located in special locations of the breast, for example, upper inner quadrant. In this article, an optimized oncoplastic technique named the “Cross” technique is introduced to overcome the abovementioned problems in the surgery of breast tumors located in the upper inner quadrant far from the center of the breast. Nineteen oncoplastic surgeries were performed by the same breast surgeon. The mean diameter and weight of the excised specimens were 20 mm and 74 g. The mean age of the patients was 51 years. Clear surgical margins were obtained in all patients. There was no marked deformity in the breast after surgery. The optimized technique produced promising results in our hands when applied to a selected group of patients. Moreover, the technique was found to reduce the need for revision surgery in ptotic breasts, as the alteration in the shape of the breast undergoing surgery is not significant enough to introduce asymmetry to the breasts.

## INTRODUCTION

Breast cancer is the most common type of malignancy and the prominent reason for cancer-related death among women worldwide.^1,2^ Even though treatment methods for this cancer have greatly evolved during the past 15 years, surgery still remains the main treatment for early breast cancer.^3^ For many years, different techniques of mastectomy were considered as the gold standard in the surgical management of this disease.^4^ The first revolution in treatment occurred with the advent of breast-conserving surgery in the 1970s in the reports published by Fisher and Veronesi.^5,6^ Breast surgery was then revolutionized with the use of plastic surgery to reshape the breast in a procedure that came to be known as oncoplastic breast surgery (OBS).^4^ These techniques are constantly being refined and upgraded as surgeons attempt to introduce new techniques with better cosmetic outcomes while always keeping in mind the oncologic safety of the procedure.^7–11^

OBS techniques are classified into “level I” and “level II” procedures. Level I OBS is performed when less than 20% of breast volume is destined to be excised. In this procedure, tissue displacement techniques are performed to repair the defect, and the nipple-areola complex (NAC) is repositioned if required.^12^ The resection of 20%–50% of the breast volume requires level II techniques. A wide range of techniques is used in level II OBS, which involves extensive skin resection with a variety of volume displacement or replacement methods. Therefore, local tissue displacement and glandular reshaping (eg, round-block, omega, vertical, radial, V-, J-, and L-type incisions) fill smaller defects.^4,13^ Furthermore, the appropriate oncoplastic technique will depend on such factors as the breast size, ptosis, and tumor size and location. For instance, for medium to large tumors in a peripheral location of a small or medium breast, oncoplastic tumorectomy is recommended.^13^

However, surgeons are faced with 2 major problems with the current span of OBS techniques. The first one concerns tumors located in some specific areas of the breast, for example, the upper inner quadrant (UIQ). This problem will be more exacerbated when the resection zone is furthest from the NAC. This means that the excision of tumors closes to the junction of the sternum and clavicle, where there is a smaller amount of breast tissue and where the surgeon should ideally avoid incisions, represent one of the most problematic surgical planning in breast-conserving surgery. The other problem is posed by the size of the breast, with smaller breasts being more challenging. In these situations, techniques in which the NAC is displaced or the skin is resected are not suitable choices.^14^

The present original article introduces a novel oncoplastic technique that we call the “Cross” technique. This technique seems to overcome most of the abovementioned problems. It was applied to a selected group of breast cancer patients with tumors located in the far UIQ of the breast. The technical details and outcomes are presented here.

## METHODS

### Patient Selection

Although the “Cross” technique can be applied to tumors located everywhere in the breast, the best candidates are patients with tumors located in the farther areas of the upper medial quadrant of the breast. Furthermore, it can be applied to different breast sizes, although it works best in patients with a medium or small breast size or patients who wish to have almost symmetrical breasts after the procedure.

The inclusion criteria were as follows: (1) having pathologically proven breast carcinoma; (2) having the tumor in the UIQ far from the NAC; (3) being a candidate for breast-conserving surgery and oncoplastic repair according to the recommendations of multidisciplinary team; and (4) giving consent to be operated on with this new technique. Patients were excluded if they had (1) any indication for mastectomy; (2) diffused ductal carcinoma in situ; (3) positive *BRCA* mutations; (4) a recurrent tumor; (5) a history of radiation therapy; and (6) preference for mastectomy or other traditional oncoplastic techniques.

### Ethical Considerations

The study was approved by the institutional review board of the Department of Surgery, Tehran University of Medical Sciences. All patients were fully informed about the new procedure at the time of surgery scheduling. They were ensured that the oncologic safety of the procedure would be the first priority and that the surgical considerations were the only concerns throughout the procedure. They were also fully informed of the potential complications of different OBS techniques and this new technique. All patients signed an informed consent before the surgery.

### Patient Preparation

**Fig. 1.**
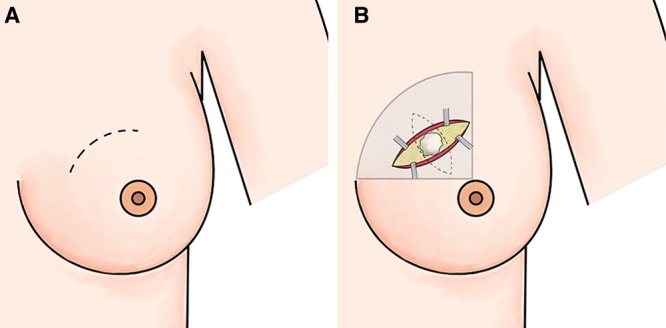
Skin incision, dissection extent and tumor resection. A, Skin marking in a curvilinear shape. The dashed line shows the skin incision line in the upper inner quadrant. B, Incision through the epidermis and dermis layers. The incised skin edges are held apart with retractors. Undermined skin area has been shown with dotted pattern and lesion excision area (breast tissue that includes the tumor) has been shown with dashed line. Removed tumor and accompanying breast tissue. Tumor and adjacent tissue are excised from subcutaneous tissue to pectoralis fascia.

The preparation of the patients for surgery was similar to the level I OBS procedures. As the first step, the exact location and the extent of the skin incision line were marked with the patient in an upright position. Skin marking was done in a curvilinear shape. It is recommended that the medial end of the incision line be terminated at a minimum of 2 cm from the lateral margin of the sternum (Fig. 1A). The operation was performed in the supine position. The arm of the patient on the operation side was abducted to allow access to the axillary region. Sterilization and draping of the operation site were done in a way that the breast and the axillary region were exposed.

The operation started with skin incision. Depending on the necessity of the resection of the skin overlying the tumor, the incision can be made in a curvilinear or an elliptical pattern. The incision line was drawn near to or on the areolar margin. If the diameter of the areola was large enough to ensure a good exposure of the surgery field, the incision line would be marked on the areolar margin. It is important to draw the line close to the areolar margin even if the tumor is located farther. Drawing or extending the line to the area of cleavage (“décolleté” in French) line should be avoided. Although the best incision in the upper part of the breast is a circum-areolar, some parts of the breast should be protected from the incision for aesthetic concerns. In other words, surgeons should avoid making an incision in the “no man’s land” area of the breast, which means above the cleavage (décolleté) line, that is, the part of the breast that might be exposed by the neckline of a woman’s clothing.

#### Procedure

##### Skin Incision and Tissue Preparation

The incision was made through the epidermis and dermis layers (Fig. 1B). This type of incision helps to preserve the skin. After the incision, the skin was separated from the underlying tissue with a thickness of 5–7 mm depending on the thickness of the skin, as in the procedure for preparing the mastectomy flaps within the areolar hypovascular plane. The extent of detachment of the skin from the breast tissue is of great significance. It should be just large enough to provide a good exposure for the tumor resection to prevent skin deformity, for example, dimpling after repair and approximation of the breast tissue following lumpectomy (See Video, [online], which displays the cross-technique OBS).

**Video 1.** This video displays the cross-technique oncoplastic breast surgery.

##### Breast Tissue Resection

**Fig. 2.**
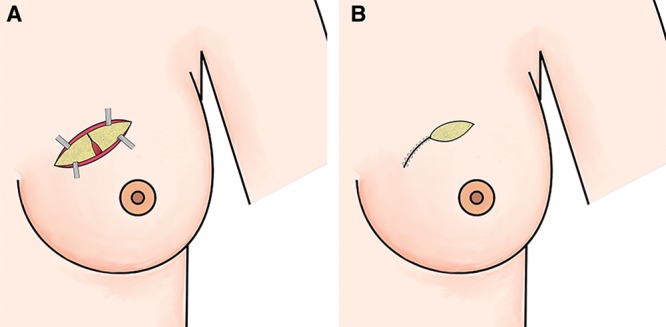
Repair of the breast tissue and the skin. A, Reapproximation and suturing of the gland. B, Skin reapproximation and suturing.

Breast tissue was resected through an elliptical breast tissue incision. The tissue resection ellipse should cross the skin incision at a right angle, hence the name “Cross”. Then, a classic lumpectomy from subcutaneous space to the prepectoral fascia was performed (Figs. 1, 2). The resection of the tumor should be performed meticulously to ensure clean margins. The repair of the resection site (elliptical tissue defect) was performed radially, followed by the placement of clips for more precise localization of the tumor site for radiation therapy.

##### Repair and Steps of the Surgery

After closing the breast tissue defect, the placement of a closed suction draining system is recommended, though is not mandatory. The procedure was finished by suturing the subcutaneous tissue and skin. Based on the preoperative plan or the intraoperative results and the decision of the multidisciplinary team, an axillary surgery (axillary lymph node dissection or sentinel lymph node biopsy) could be performed through a separate incision.

## RESULTS

**Fig. 3.**
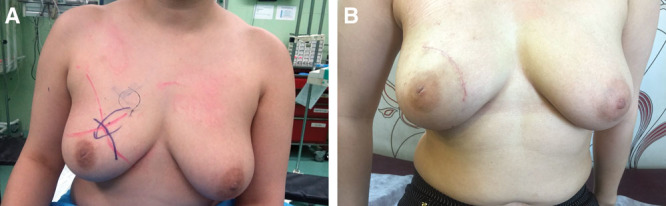
Photographs of a random patients before (A) and after (B) the surgery.

Nineteen oncoplastic surgeries were performed by the same breast surgeon from January 2016 to March 2018. All patients had tumors located in the UIQ of the breast. The mean age of the patients was 51 years, and most patients had an underbust girth of 80–85 cm (9 patients with brassiere cup size B and 10 with size C). The mean diameter and weight of the excised specimens were 20 mm and 74 g. Clear surgical margins were obtained in all patients and a closed suction draining system was placed during the procedure. There was no marked deformity or asymmetry in the breasts of the patients after the operation. An average of 6 lymph nodes was excised, and 4 patients were found to have node involvement. Twelve patients were diagnosed with invasive ductal carcinoma and ductal carcinoma in situ; other patients were diagnosed with either invasive ductal carcinoma or atypia. None of the patients presented with early postoperative complications (hematoma or infection) or delayed complications (fat necrosis or breast tissue ischemia). During the mean follow-up of 5 months, none of the patients developed local recurrence or required a second surgery. Before-and-after photographs of 2 random patients are shown in Figure 3.

## DISCUSSION

Theoretically, when a tumor happens to be in the spectrum of classic surgical patterns, its removal should pose great difficulty. In a recent study, 51.5% of tumors happened to be in the upper outer quadrant, whereas 15.6%, 14.2%, 10.6%, and 8.1% of tumors were located in the UIQ, lower outer quadrant, center, and lower inner quadrant of the breast, respectively.^15^ Studies on the incidence of breast cancer in various breast quadrants show that the far-zone tumors are those that occur in the upper part of the breast.^16^ They can be located in all parts of the upper half of the breast, both laterally and medially. Although numerous OBS techniques have been introduced for tumors in these locations, each has its own difficulties and problems.^7–9,17^ However, regarding tumors in the UIQ, oncoplastic breast surgeons are faced with several problems, especially when the tumor is located far from the NAC.

Surgical plan for resection of the tumors in the UIQ of the breasts is more problematic because this area is aesthetically important to women. That is why there is a critical need for new methods that are able to avoid the disadvantages of the current techniques. Our results showed that the “Cross” method has several advantages that can make it a suitable alternative to the classical methods of surgery for tumors in the UIQ, far from the NAC.

Breast size is an important issue in oncoplastic surgery. In patients with relatively large breasts, level II (reduction type) OBS would be the preferred choice in many situations. However, in cases where the patient would not like to change the size of her breasts or lose the symmetry of her breasts or does not wish to undergo a major operation, level I OBS should be applied. In these situations, and in patients with medium-sized breasts, the residual tissue in the volume displacement techniques is usually sufficient to obtain satisfactory cosmetic outcomes. Even in these conditions, the “Cross” technique could be the preferred choice as it minimizes the risk of asymmetry and the scale of the operation. In contrast, in small or even medium breasts (where the volume of the resection relative to the breast is large), planning an oncoplastic surgery with limited residual volume seems to be a challenge.^18^ One of the key features of the “Cross” method is that it provides satisfactory cosmetic outcomes in small breasts.

OBS techniques aim to prevent the breast deformity after the resection of the tumor in the breast, although the rate of breast deformity is still relatively high in some situations.^19^ The location of the tumor is one of the main factors affecting deformity after lumpectomy. Even with the improvement of the oncoplastic surgery techniques, the chance of breast deformity or breast or NAC asymmetry is considerably high when the tumor is located in the UIQ. The first prominent advantage of the new technique is to avoid making the incision where it is aesthetically important. A conventional tumorectomy in the UIQ would usually leave a visible scar and the patient would have to wear clothes to cover the incision line, whereas, in the suggested technique, the incision is made below cleavage (décolleté in French) and is not visible. On the other hand, conducting surgery on a tumor that lies far from the NAC is, inevitably, more difficult.

Several techniques have been introduced for each breast quadrant. Most experts believe that the upper part of the breast and the UIQ are the least favorable locations especially in a situation that the tumor is far from the center of the breast.^20^ The Cross method makes it possible to conduct the surgery in the far zones of the UIQ even in small breasts, where other types of surgery would not offer an acceptable cosmetic outcome. The absence of any postoperative deformity in the patients in this study supports the abovementioned claim.

Traditionally, a tumor close to the skin has been considered a contraindication for performing breast-conserving therapy. However, recent data show that these patients can be good candidates for breast-conserving surgery after neoadjuvant chemotherapy. A common problem with the conventional breast-conserving techniques is that the skin incision needs to be made over the tumor and overlying involved skin. The new technique, however, offers the flexibility of making the incision farther from the tumor (namely, under cleavage) unless there is skin involvement or any other indication for skin resection.

In a study, a clear majority of patients (>80%) who underwent OBS stated that they would make the same choice again if required.^21^ Compared with classical procedures, patients are more satisfied with the aesthetic results of OBS and have higher rates of self-esteem.^22^ However, in comparison with classic conservative treatment, patients who undergo oncoplastic surgery have higher expectations,^23^ which is mostly due to the lower rates of breast deformity after OBS. We did not detect any marked deformity in any patient. The right angle between the breast tissue incision and the skin incision prevents changing the breast shape and, more importantly, the place of the NAC, especially in visible areas of the breast (UIQ). On the other hand, when the incision of the skin and the breast tissue are made in the same direction, the scar tissue and healing process will cause marked adhesion and contraction that begins from the skin to the pectoral muscle level in the same direction. This causes a “fold shape” in the upper part of the breast while the right angle incision of the skin and breast tissue can easily prevent this problem. Indeed, by using the Cross method, the parenchymal tissue will be approximated by sutures in a direction that prevents the folding and linear dimpling of the breast in the operation site.

Regarding the surgical complications, as with other OBS techniques, the risk is quite low with the new technique, which is due to the single incision line and minimal manipulation of the breast tissue. Therefore, applying the Cross method would provide the patients with a quick recovery from the procedure.

A critical issue concerning the current OBS techniques is the variety of techniques. The choice of the appropriate technique varies with the location of the tumor, which can particularly be confusing for the young surgeons. Whereas the classical techniques are complicated and require huge time and effort to learn and master, the “Cross” methods are easy to learn and need a shorter operation time.

In breast-conserving surgery, there is always a risk of incomplete resection and involved margins. When postoperative pathological evaluation identifies a positive margin, reexcision and additional resections are indicated. The rate of identifying positive margins after OBS has been reported to be 7%–12%.^24–26^ Because the displacement of breast tissue in most cases of OBS is considerable, the determining of the exact margin involved represents a challenge in reexcision of the margins in these patients. Nevertheless, in the introduced technique, if additional surgery was needed to obtain clear margins, reopening the breast and reexcision of the margins would not be a great deal.

## CONCLUSIONS

Based on our results and on the theoretical and technical points discussed here, we would recommend breast surgeons to use the “Cross” technique in breast-conserving reconstruction surgeries. The best indication for this technique is a tumor located in the UIQ of the breast. The indication becomes stronger as the tumor location gets farther from the NAC, especially in medium and small breasts.

## Acknowledgment

The authors wish to thank Ms. Parastoo Saberi of Tehran University of Medical Sciences who designed the artworks and figures of the article.
